# Simple, Axial Ligand-Mediated Route to Water-Soluble Iridium Corroles

**DOI:** 10.1021/acsomega.1c02399

**Published:** 2021-06-15

**Authors:** Ivar K. Thomassen, Daniel Rasmussen, Rune F. Einrem, Abhik Ghosh

**Affiliations:** Department of Chemistry, UiT—The Arctic University of Norway, N-9037 Tromsø, Norway

## Abstract

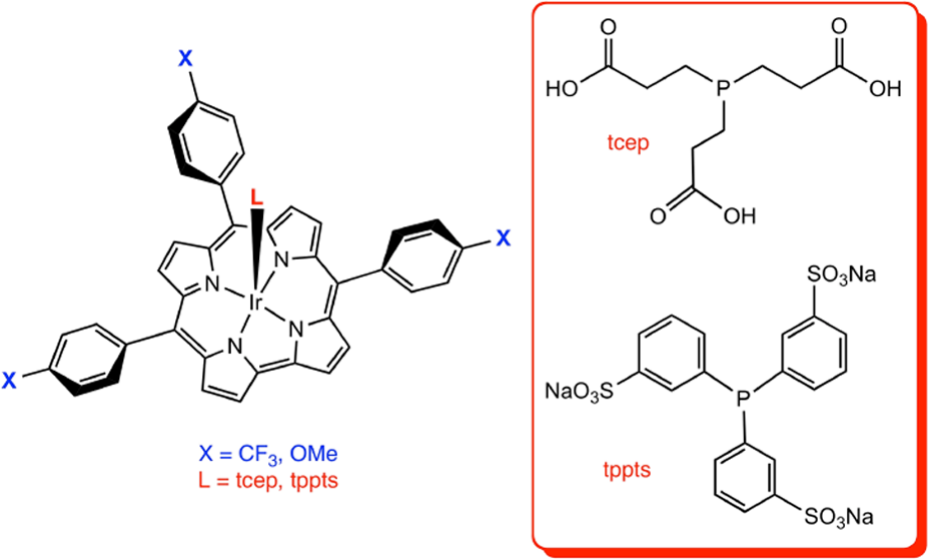

The synthesis and purification of water-soluble porphyrin-type compounds for photodynamic therapy and other medical applications is often a tedious exercise. Here, we have investigated the simple stratagem of adding a water-soluble axial ligand to the standard protocol for iridium insertion into simple *meso*-triarylcorroles. Early results showed that six-coordinate Ir[T*p*XPC](dna)_2_ derivatives, in which T*p*XPC = tris(*para*-X-phenyl)corrole (X = CF_3_, CN, H, and OMe) and dna = dinicotinic acid, are highly water-soluble. In the end, however, all axially nitrogen-ligated complexes proved unstable with respect to chromatographic purification and storage. Five-coordinate water-soluble phosphine adducts, fortunately, proved a great improvement. From the point of view of ease of purification and storage, the best products proved to be Ir[T*p*XPC](L), where X = CF_3_ and OMe and L = tris(2-carboxyethyl)phosphine (tcep) and trisodium tris(3-sulfonatophenyl)phosphine (tppts); carefully optimized synthetic protocols are presented for these four compounds.

## Introduction

**Scheme 1:**
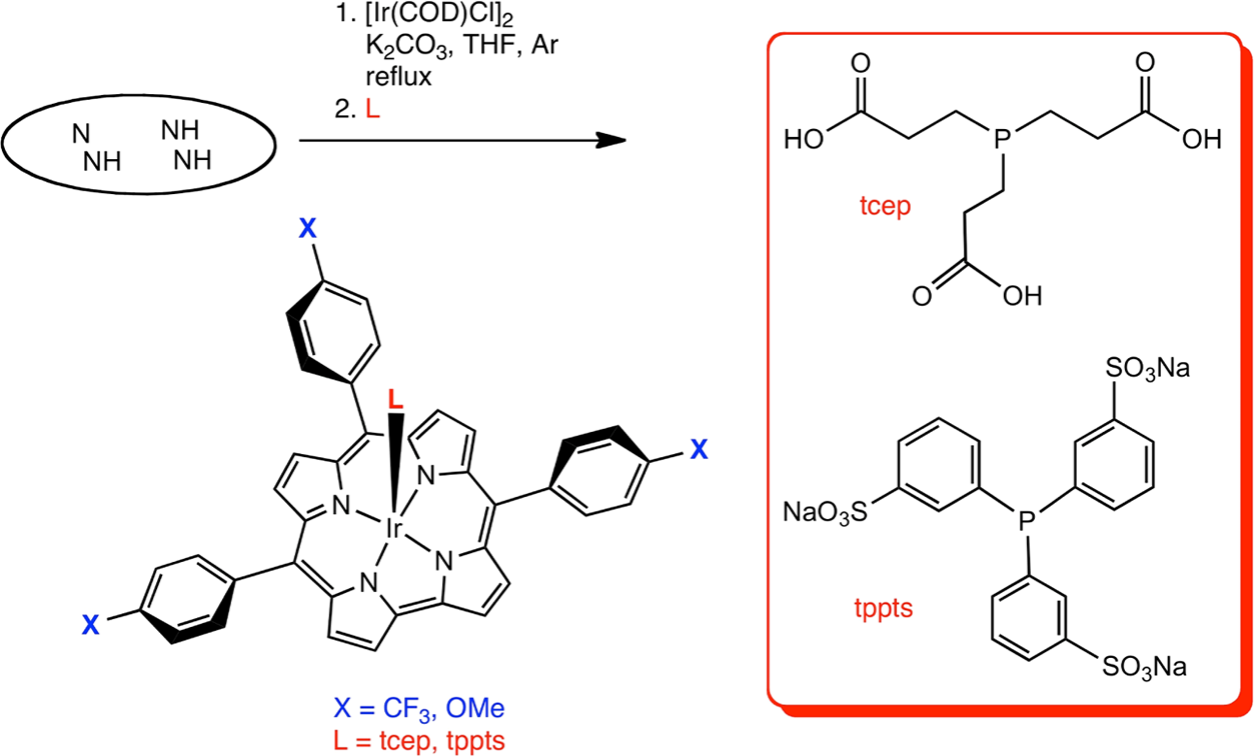
Synthesis of Four Stable, Water-Soluble Iridium Corroles

Porphyrin-type compounds have long been a cornerstone of photodynamic therapy.^1−5^ Recently, porphyrin analogues such as corroles^6,7^ have also proved promising as anticancer compounds.^8,9^ Several families of 5d metallocorroles (including ReO,^10^ OsN,^11^ Ir,^12^ Pt,^13^ and Au^14−17^ corroles) that we and others have studied in recent years are relevant in this connection. Although they were originally of interest primarily as curious, size-mismatched metal–ligand assemblies, their photophysical properties, especially near-infrared (NIR) phosphorescence under ambient conditions, now promise a wide range of practical applications,^18,19^ such as in oxygen sensors, photodynamic therapy, and dye-sensitized solar cells and for triplet–triplet annihilation upconversion.^20−28^ A number of these applications, especially in the biomedical sphere, require water-soluble derivatives of the complexes, which are typically accessible via cumbersome synthetic and purification steps.^2,29,30^ A recent reinvestigation of iridium corroles (in which 4-picolinic acid derivatives were found to be partially water-soluble) suggested that the use of water-soluble axial ligands might afford a simple, one-pot route to water-soluble Ir corroles as a new class of singlet oxygen photosensitizers.^31^ The beguilingly simple exercise, however, threw up unexpected challenges. Many of the compounds synthesized proved unstable, decomposing upon chromatographic purification or storage. Here, we detail carefully optimized synthetic protocols for four complexes (Scheme 1) that could be readily purified and stored and are therefore suitable for further investigations of potential applications.

## Results and Discussion

**Figure 1:**
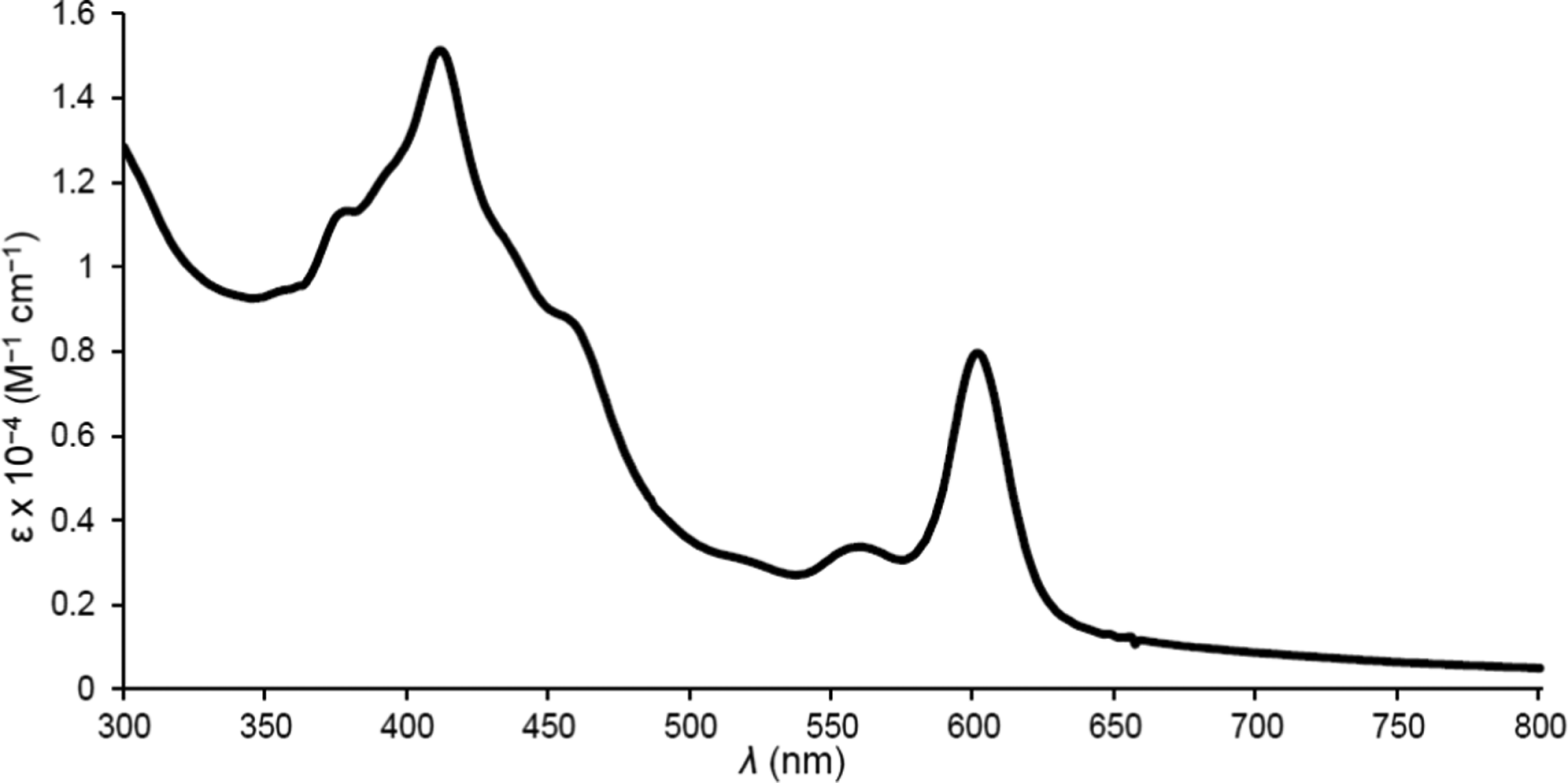
UV–vis spectrum of Ir[T*p*OMePC](4pa)_2_ in methanol.

**Figure 2:**
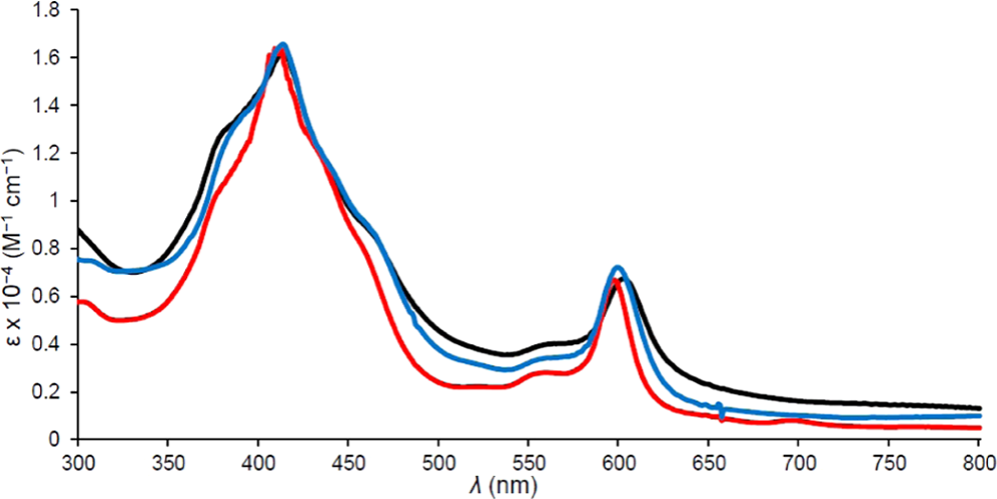
UV–vis comparison of freshly prepared Ir[T*p*OMePC](dna)_2_ (black), Ir[TPC](dna)_2_ (red), and Ir[T*p*CF_3_PC](dna)_2_ (blue) in methanol.

**Figure 3:**
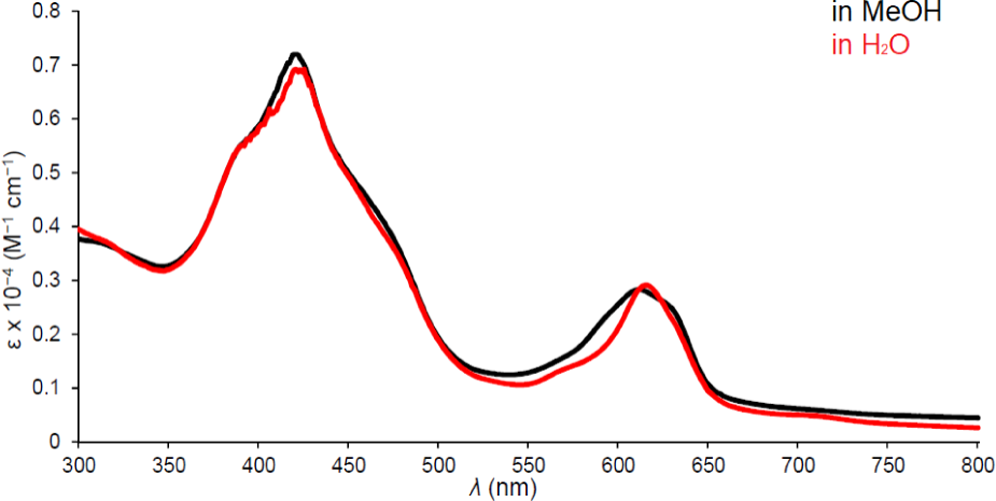
UV–vis spectrum of Ir[T*p*CF_3_PC](tcep) in methanol and water.

Six different *meso*-tris(*para*-X-phenyl)corrole ligands, H_3_[T*p*XPC] (X = NO_2_, CF_3_, CN, H, Me, and OMe),^32−34^ as well as *meso*-tris(pentafluorophenyl)corrole, H_3_[TPFPC],^35^ were examined throughout as equatorial ligands. For axial ligands, we initially examined five nitrogen ligands—4-picolinic acid (4pa; Figure 1), 3,5-pyridinedicarboxylic acid (also known as dinicotinic acid, dna), nitrilotriacetic acid (nta), 5-hydroxypyridine-3-carboxylic acid, and 4-pyridylboronic acid. Four different stationary phases were used for chromatographic purification of the complexes—silica gel, basic and neutral alumina, Florisil, and fully endcapped C_18_ reversed-phase silica gel. Although iridium insertion could be accomplished for all of the corroles except X = NO_2_, the great majority of the complexes proved unstable; bright green solutions of the freshly prepared complexes frequently turned brown, often with the decomposition product sticking to the glass walls of the reaction vessel. The most promising of the lot proved to be the dna complexes Ir[T*p*XPC](dna)_2_ (X = CF_3_, CN, H, and OMe; Figure 2), exhibiting high water solubility, but these too proved unstable upon chromatographic workup and/or storage, as indicated by the disappearance of the highly characteristic optical spectra. Attempts to avoid chromatography by resorting to solvent extraction and vacuum filtration ultimately also proved unsuccessful.

**Figure 4:**
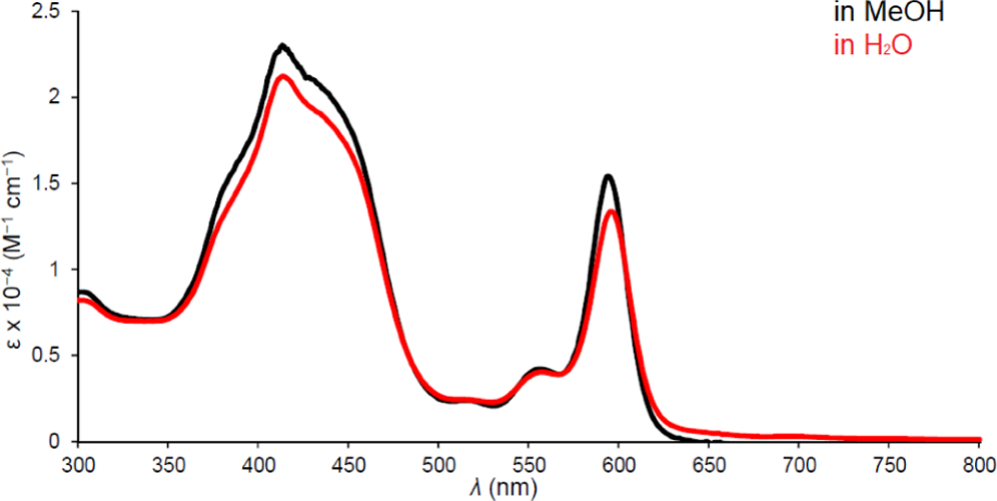
UV–vis spectrum of Ir[T*p*CF_3_PC](tppts) in methanol and water.

**Figure 5:**
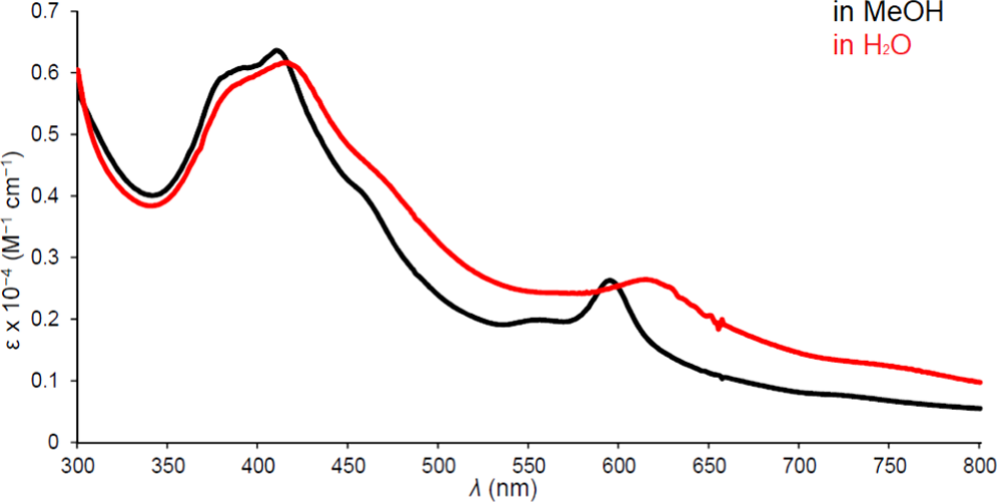
UV–vis spectrum of Ir[T*p*OMePC](tcep) in methanol and water.

Mass spectrometric analyses of the decomposed complexes generally revealed large quantities of free axial ligands, suggesting that they tend to fall off during chromatographic purification. This observation led us to switch to water-soluble phosphine ligands,^36^ of which we examined three—tris(2-carboxyethyl)phosphine (tcep), trisodium tris(3-sulfonatophenyl)phosphine (tppts), and tris(hydroxymethyl)phosphine (thp). Like triphenylphosphine,^37^ these phosphine ligands also led to five-coordinate complexes, which fortunately also proved distinctly more stable than the nitrogen-ligated complexes described above. Of the phosphine complexes, the thp derivatives proved poorly soluble in water (presumably reflecting the lower hydrophilicity of the alcohol functionality relative to carboxylate and sulfonate) and were accordingly excluded from further examination in our study. Fortunately, the tcep and tppts complexes proved fully soluble in distilled water (see Figures 3–5 and Table 1 for representative optical spectra and spectral data). A number of tcep complexes (especially for X = CN, H, and Me), however, proved somewhat hygroscopic, and the optical spectra exhibited broadening upon prolonged standing in water. In contrast, Ir[T*p*CF_3_PC](tcep) proved unusually rugged, remaining unchanged in air and both aqueous and nonaqueous solutions for days. Finally, the tppts complexes proved highly stable (albeit slightly hygroscopic, thereby thwarting our attempts at obtaining accurate elemental analyses) as well as readily purifiable via column chromatography on regular silica gel without any problems.

**Table 1:** Absorption Maxima (λ, nm) for Ir Corroles^a^

complex	solvent	*B*	*Q*
Ir[T*p*OMePC](tcep)	MeOH	389, 411*	609*
Ir[T*p*OMePC](tppts)	MeOH	391, 413*	559, 594*
Ir[T*p*CF_3_PC](tcep)	MeOH	420*	610*
Ir[T*p*CF_3_PC](tppts)	MeOH	413*, 427	556, 594*
Ir[T*p*OMePC](tcep)	H_2_O	416*	615*
Ir[T*p*CF_3_PC](tcep)	H_2_O	425*	615*
Ir[T*p*CF_3_PC](tppts)	H_2_O	413*	557, 595*

a The numbers marked with an asterisk indicate the wavelengths with the most intense absorption.

A brief word on the optical spectra of the new compounds may be of interest. The spectra (Figures 1–5) clearly show highly distinctive absorption profiles, including a complex Soret manifold and a *Q* manifold, whose shape also varies considerably. Thus, the sharp and intense *Q* band of Ir[T*p*CF_3_PC](tppts) (Figure 4) may be distinguished from those of the tcep complexes (Figures 3 and 5). These distinctive spectra provided simple “spectroscopic handles” for assessing the integrity and purity of the compounds studied. A discussion of the electronic origin of the diverse absorption profiles, while of significant theoretical interest, is outside the scope of this study.

A couple of phosphine complexes exhibited a small quantity of impurity in their NMR spectra (see Figures S9–S16 in the Supporting Information); we have not identified this species as of yet but view a six-coordinate water or solvent adduct as a plausible candidate.

## Conclusions

The present study was motivated by a desire to synthesize water-soluble iridium corroles for photodynamic therapy and other biomedical applications via the simple stratagem of employing a water-soluble axial ligand. Although ultimately successful, the exercise entailed unexpected challenges. Thus, the complexes with axial amine ligands such as 4-picolinic acid and dinicotinic acid proved unstable, decomposing over hours to days upon standing in water. Five-coordinate phosphine complexes, in contrast, proved much more stable and readily purifiable with reversed-phase column chromatography, with tcep and tppts emerging as the most promising axial ligands. Of the various complexes synthesized, Ir[T*p*CF_3_PC](tppts) is arguably the most attractive, considering its high water solubility, long-term stability in solution, and distinctive optical signature. The performance of the new compounds in photocytotoxicity measurements is currently under evaluation and will be reported in due course.

## Experimental Section

### Materials

Tris(2-carboxyethyl)phosphine hydrochloride (99%), trisodium tris(3-sulfonatophenyl)phosphine (<10% phosphine oxide), and tris(hydroxymethyl)phosphine (95%) were purchased from Strem Chemicals, Inc. Free-base corroles were prepared as previously reported.^33−35^ Unless otherwise mentioned, all other chemicals were obtained from Sigma-Millipore (Merck).

### Instrumental Methods

UV–visible spectra were recorded on an HP 8453 spectrophotometer. ^1^H NMR, ^19^F NMR, and ^31^P NMR spectra were recorded on a 400 MHz Bruker Avance III HD spectrometer equipped with a 5 mm BB/1H SmartProbe in CD_3_OD or (CD_3_)_2_SO. ^1^H NMR spectra were referenced to residual CH_3_OH (3.31 ppm) or to (CH_3_)_2_SO (2.50 ppm). High-resolution electrospray-ionization (HR-ESI) mass spectra were recorded from methanolic solution on an LTQ Orbitrap XL spectrometer.

### General Procedure for the Synthesis of Ir[T*p*XPC](L) (X = OMe, CF_3_; L = tcep, tppts)

The iridium complexes were prepared according to a modified version of a previously reported procedure.^12^ Bis(1,5-cyclooctadiene)diiridium(I) dichloride (1.5 equiv) and potassium carbonate (10 equiv) were dissolved in an anhydrous tetrahydrofuran (THF) solution (20 mL) of a free-base corrole (∼0.025–0.1 mmol, 1 equiv). After degassing with argon for a few minutes, the solution was brought to reflux under an inert atmosphere. Heating was discontinued after 90 min, and the phosphine (1 equiv) was added as a solution of anhydrous methanol (10 mL); the reaction mixture was then left to stir for 30 min. As alluded to above, four products were purified and fully characterized, as described below. Because of the hygroscopic nature of the compounds, satisfactory elemental analyses, in general, could not be obtained; the yields accordingly should be regarded as upper limits.

#### Ir[T*p*OMePC](tcep)

The reaction mixture was rotary-evaporated to dryness. The dark solid residue was suspended in dichloromethane and thoroughly shaken; the solvent was decanted off to remove unreacted free-base corrole and other nonpolar impurities. This step was repeated with ethyl acetate and acetonitrile, finally leaving behind a dark green solid, which was dissolved in methanol. The solution was filtered to remove any remaining salts, and the filtrate was evaporated to dryness. The resulting solid was dissolved in a minimum amount of methanol and chromatographed on a fully C_18_-endcapped reversed-phase silica gel column with different MeCN/MeOH mixed solvents as the mobile phase (as detailed below), yielding the expected product along with some silica particles as a light green solid. The silica particles were removed by suspending the solid in pentane, sonicating the suspension briefly, and filtering off the pentane solution containing the dissolved/micro-suspended silica. The dark green solid residue was dissolved in methanol and transferred to a new vessel; upon removal of the solvent under vacuum, the product was obtained as a deep green, hygroscopic solid. Yield 98 mg (85%). UV–vis (CH_3_OH) λ_max_ (nm) [ϵ × 10^–4^ (M^–1^ cm^–1^)]: 389 (sh, 0.56), 411 (0.62), 609 (0.25). ^1^H NMR (400 MHz, methanol-*d*_4_) δ 8.63 (d, *J* = 4.0 Hz, 2H), 8.48 (d, *J* = 4.0 Hz, 2H), 8.29 (d, *J* = 4.7 Hz, 2H), 8.09 (t, *J* = 4.8 Hz, 2H), 7.91–7.80 (m, 4H), 7.32–7.14 (m, 8H), 4.00 (d, *J* = 4.6 Hz, 9H), −0.19 (t, *J* = 8.2 Hz, 6H), −1.58 (t, *J* = 8.2 Hz, 6H). ^31^P NMR (162 MHz, composite pulse-decoupled, methanol-*d*_4_) δ −22.43. MS (ESI): [M^–^] = 1055.2415 (expt), 1055.2406 (calcd for IrC_49_H_43_N_4_O_9_P).

#### Ir[T*p*OMePC](tppts)

The reaction mixture was rotary-evaporated to dryness, yielding a dark green solid. The residue was dissolved in a minimum amount of methanol and subjected to column chromatography (regular silica gel, 10:1 MeCN/MeOH, then 4:1 MeCN/MeOH) to obtain the title compound as a dichroic brown-green solid. Yield 16.6 mg (22.5%). UV–vis (CH_3_OH) λ_max_ (nm) [ϵ × 10^–4^ (M^–1^ cm^–1^)]: 391 (sh, 1.82), 413 (2.02), 559 (sh, 0.72), 594 (0.84). ^1^H NMR (400 MHz, DMSO-*d*_6_) δ 7.94 (d, *J* = 1.8 Hz, 4H), 7.83 (dt, *J* = 7.6, 1.5 Hz, 4H), 7.52 (t, *J* = 7.6 Hz, 6H), 7.48–7.43 (m, 6H), 4.19–3.93 (m, 9H), 3.38 (t, *J* = 6.4 Hz, 3H), 2.23 (t, *J* = 7.4 Hz, 3H), 1.63 (p, *J* = 6.9 Hz, 3H), 1.29–1.17 (m, 3H). ^31^P NMR (162 MHz, composite pulse-decoupled, DMSO-*d*_6_) δ 26.36. MS (ESI): [M^–^] = 1328.1142 (expt), 1328.1154 (calcd for IrC_58_H_44_N_4_O_12_S_3_PNa).

#### Ir[T*p*CF_3_PC](tcep)

The purification was carried out as for Ir[T*p*OMePC](tcep) to afford the title compound as a deep green, hygroscopic solid. Yield 26 mg (90%). UV–vis (CH_3_OH) λ_max_ (nm) [ϵ × 10^–4^ (M^–1^ cm^–1^)]: 420 (0.70), 610 (0.26). ^1^H NMR (400 MHz, methanol-*d*_4_) δ 8.73 (t, *J* = 3.8 Hz, 2H), 8.62 (d, *J* = 7.9 Hz, 4H), 8.55 (d, *J* = 7.9 Hz, 2H), 8.48 (d, *J* = 4.8 Hz, 2H), 8.33 (dd, *J* = 4.8, 1.5 Hz, 2H), 8.13–8.11 (m, 2H), 7.98 (d, *J* = 8.0 Hz, 4H), 7.94 (d, *J* = 8.0 Hz, 2H), −0.28 to −0.33 (m, 6H), −2.13 (s, 6H). ^19^F NMR (377 MHz, methanol-*d*_4_) δ −63.27. ^31^P NMR (162 MHz, composite pulse-decoupled, methanol-*d*_4_) δ −22.66. MS (ESI): [M^–^] = 1169.1697 (expt), 1169.1710 (calcd for IrC_49_H_34_N_4_F_9_O_6_P).

#### Ir[T*p*CF_3_PC](tppts)

The reaction mixture was rotary-evaporated to dryness, yielding a dark green solid. The residue was suspended in dichloromethane and shaken thoroughly, and the solvent was decanted off to remove unreacted free-base corrole and other less polar impurities. The solid residue was dissolved in a minimum amount of methanol and subjected to column chromatography (regular silica gel, 5:1 CH_2_Cl_2_/MeOH, then 1:1 CH_2_Cl_2_/MeOH), affording the product as a dichroic red-green solid. Yield 26 mg (52%). UV–vis (CH_3_OH) λ_max_ (nm) [ϵ × 10^–4^ (M^–1^ cm^–1^)]: 413 (2.68), 427 (sh, 2.47), 556 (sh, 0.49), 594 (1.79). ^1^H NMR (400 MHz, methanol-*d*_4_) δ 8.64 (d, *J* = 4.3 Hz, 2H), 8.43 (d, *J* = 4.8 Hz, 2H), 8.16 (d, *J* = 4.8 Hz, 2H), 8.03–7.95 (m, 4H), 7.90 (d, *J* = 4.3 Hz, 2H), 7.88–7.51 (m, 8H), 7.39–7.34 (m, 3H), 6.76 (t, *J* = 7.8 Hz, 3H), 5.33 (d, *J* = 1.8 Hz, 3H), 3.89 (dd, *J* = 7.9, 1.6 Hz, 3H). ^19^F NMR (377 MHz, methanol-*d*_4_) δ −63.30. ^31^P NMR (162 MHz, composite pulse-decoupled, methanol-*d*_4_) δ −25.59. MS (ESI): [M^–^] = 1421.0710 (expt), 1421.0716 (calcd for IrC_58_H_34_N_4_F_9_O_9_S_3_P).

## Supporting Information Available

UV–vis, HR-ESI mass, and ^1^H NMR spectra (PDF)
