# Supplementary material for: Simple, Axial Ligand-Mediated Route to Water-Soluble Iridium Corroles
Source: ACS Omega. 2021 Jun 15;6(25):16683–7. doi: 10.1021/acsomega.1c02399 (PMC8246702; doi:10.1021/acsomega.1c02399)
Supplement: Supplementary file 1 — ao1c02399_si_001.pdf [file ao1c02399_si_001.pdf]

A Simple, Axial Ligand-Mediated Route to Water-Soluble Iridium Corroles

Ivar K. Thomassen,[a] Daniel Rasmussen,[a] Rune F. Einrem[a] and Abhik Ghosh*,[a]

Department of Chemistry, UiT – The Arctic University of Norway, N-9037 Tromsø, Norway; Email: abhik.ghosh@uit.no.

Contents **Page**

A. UV-vis spectra S2
B. ESI mass spectra S4
C. NMR spectra S10

A. UV-Vis spectra

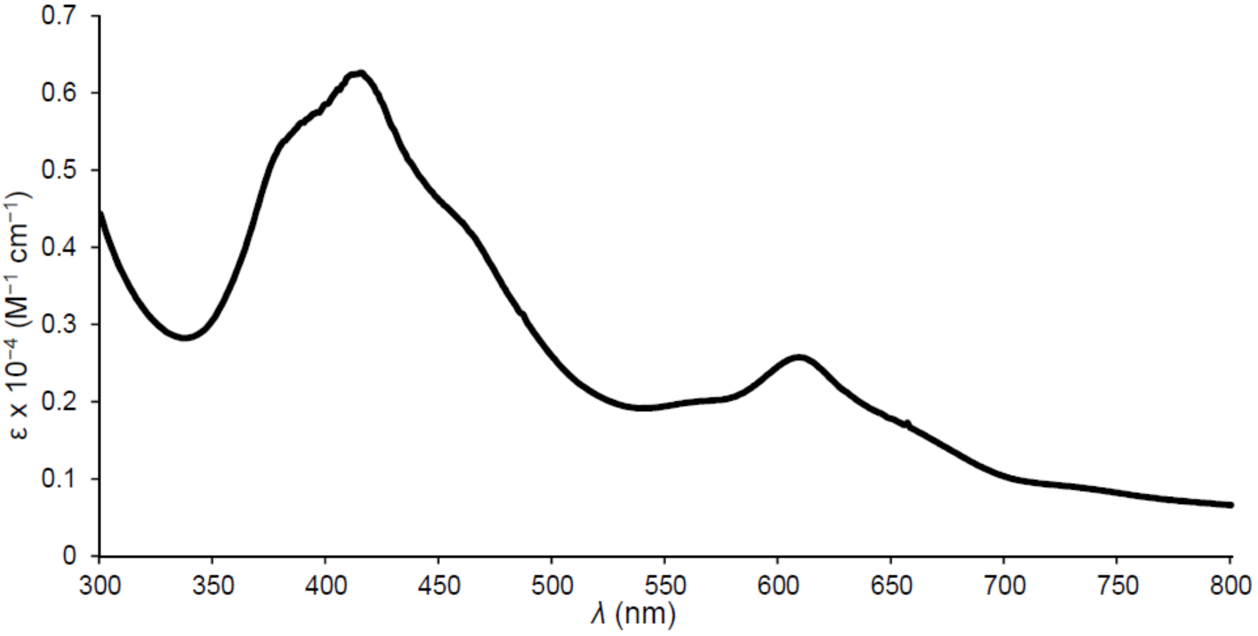

Figure S1. UV-vis spectrum of Ir[(T*p*OMePC)](tcep) in methanol.

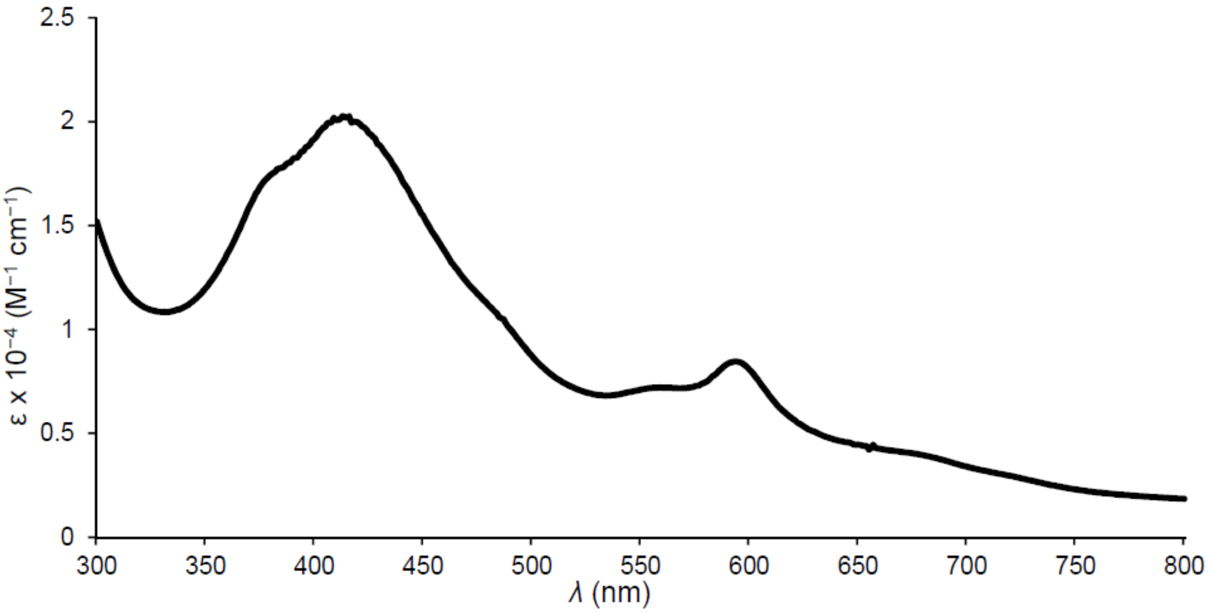

Figure S2. UV-vis spectrum of Ir[T*p*OMePC](tppts) in methanol.

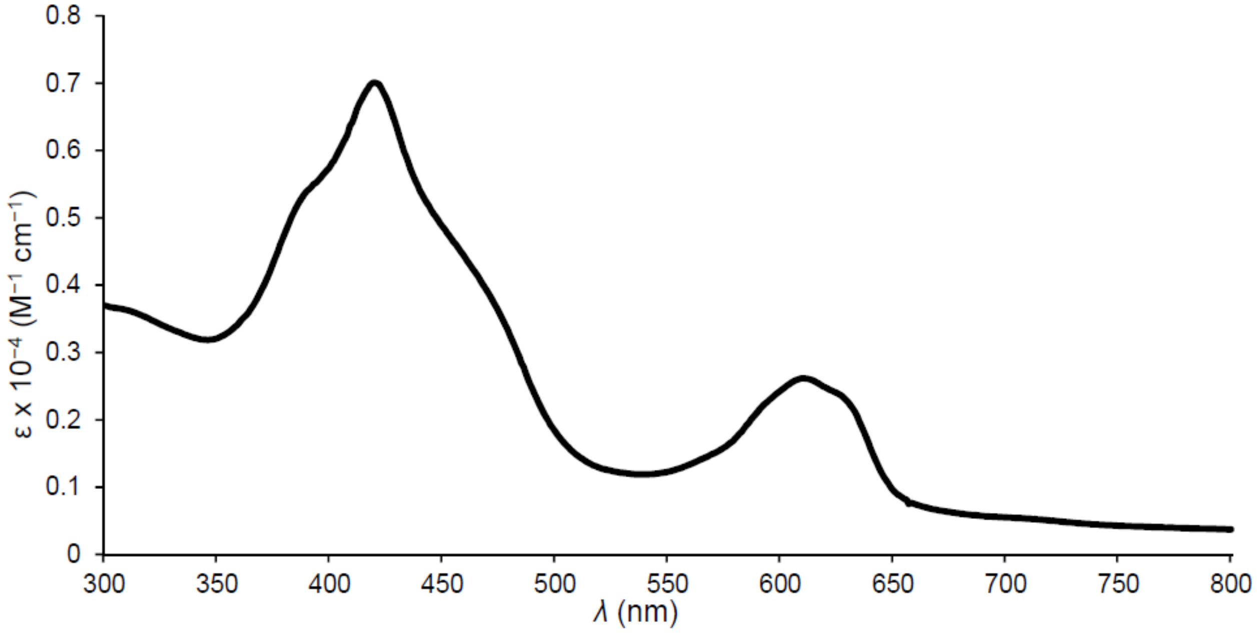

Figure S3. UV-vis spectrum of Ir[T*p*CF_3PC](tcep) in methanol.

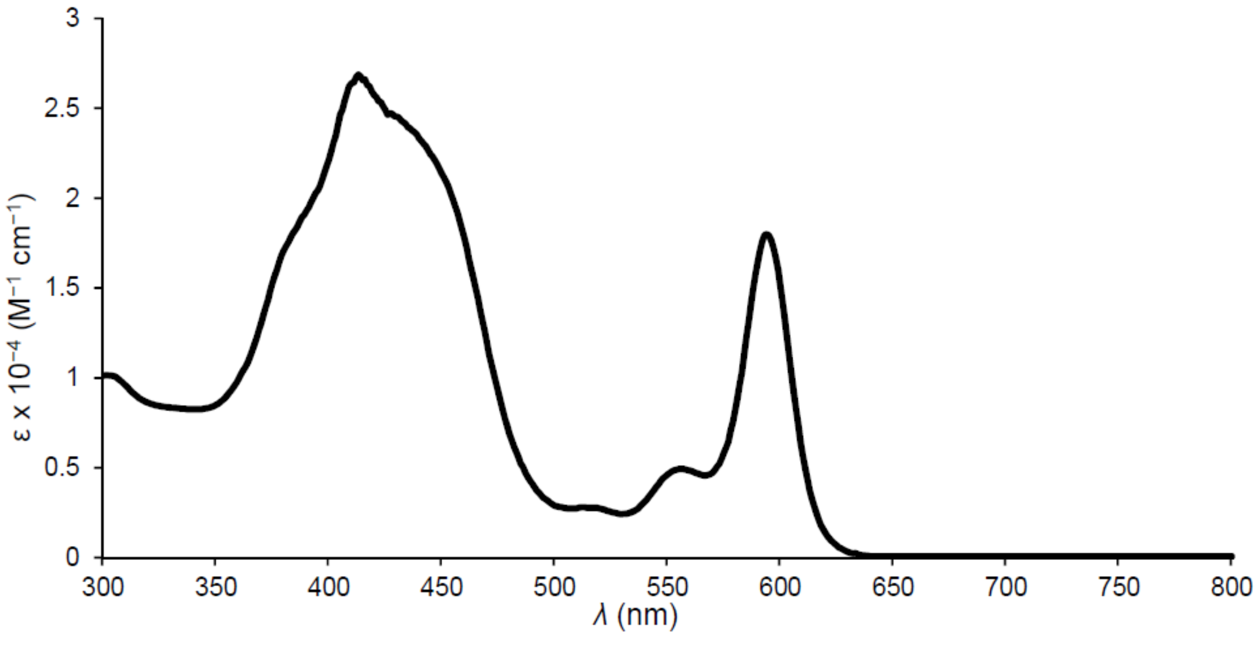

Figure S4. UV-vis spectrum of Ir[T*p*CF_3PC](tppts) in methanol.

B. ESI Mass spectra

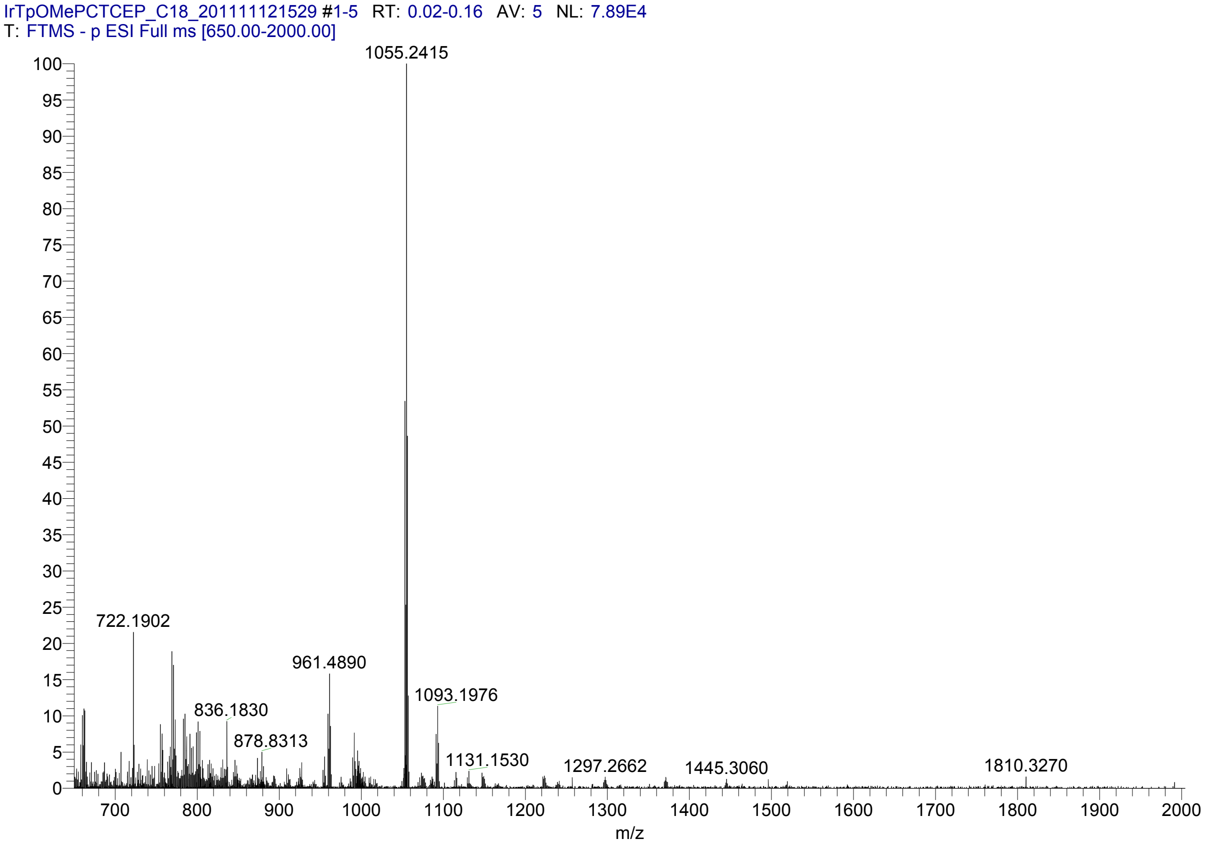

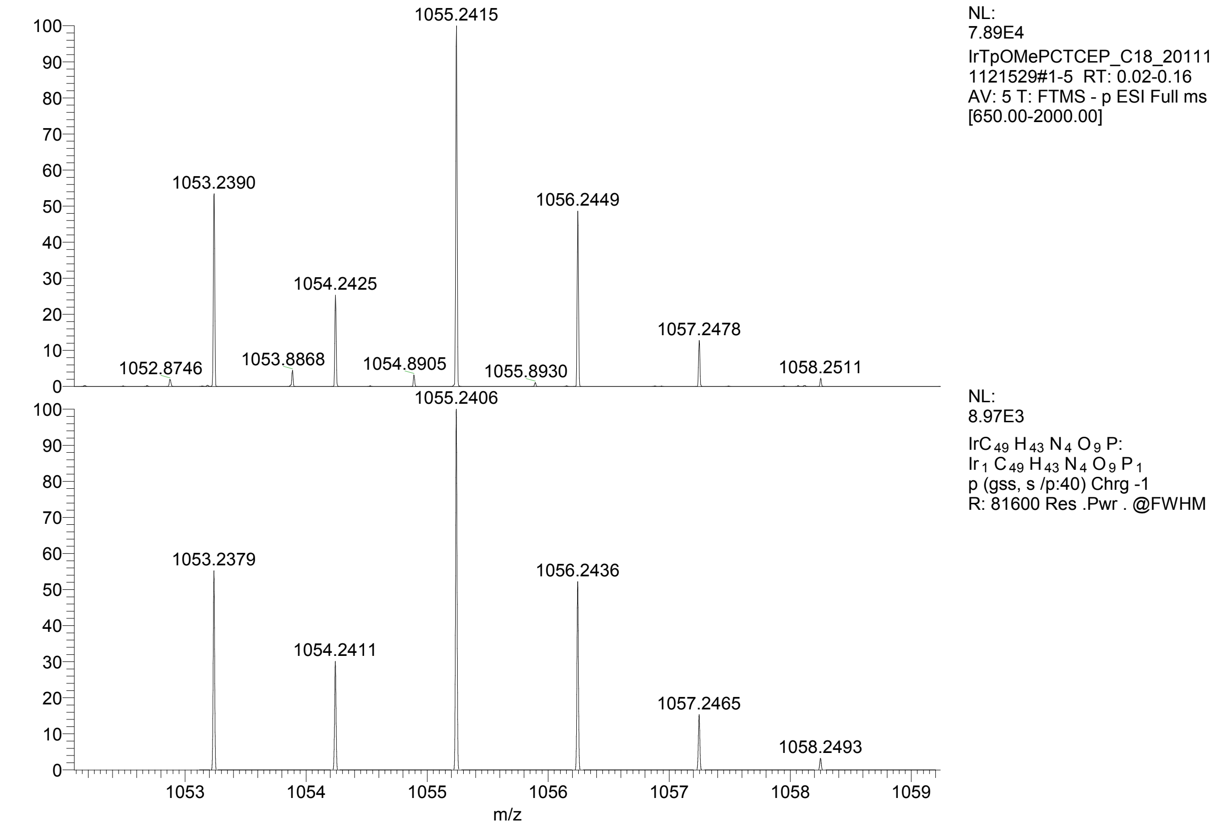

Figure S5. Negative-mode ESI mass spectra of Ir[T*p*OMePC](tcep).

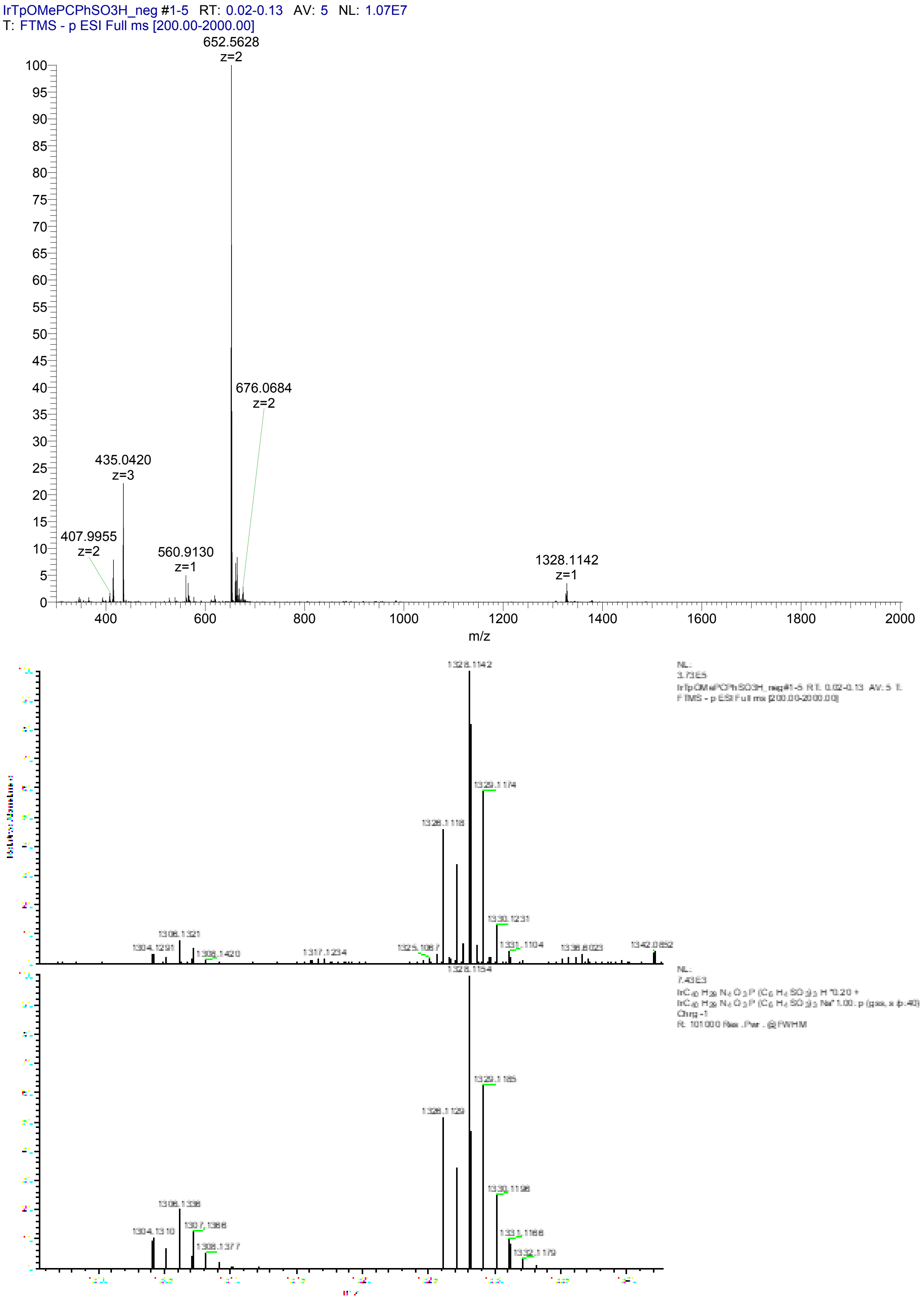

Figure S6. Negative-mode ESI mass spectra of Ir[T*p*OMePC](tppts) (Z=1). *Contd.*

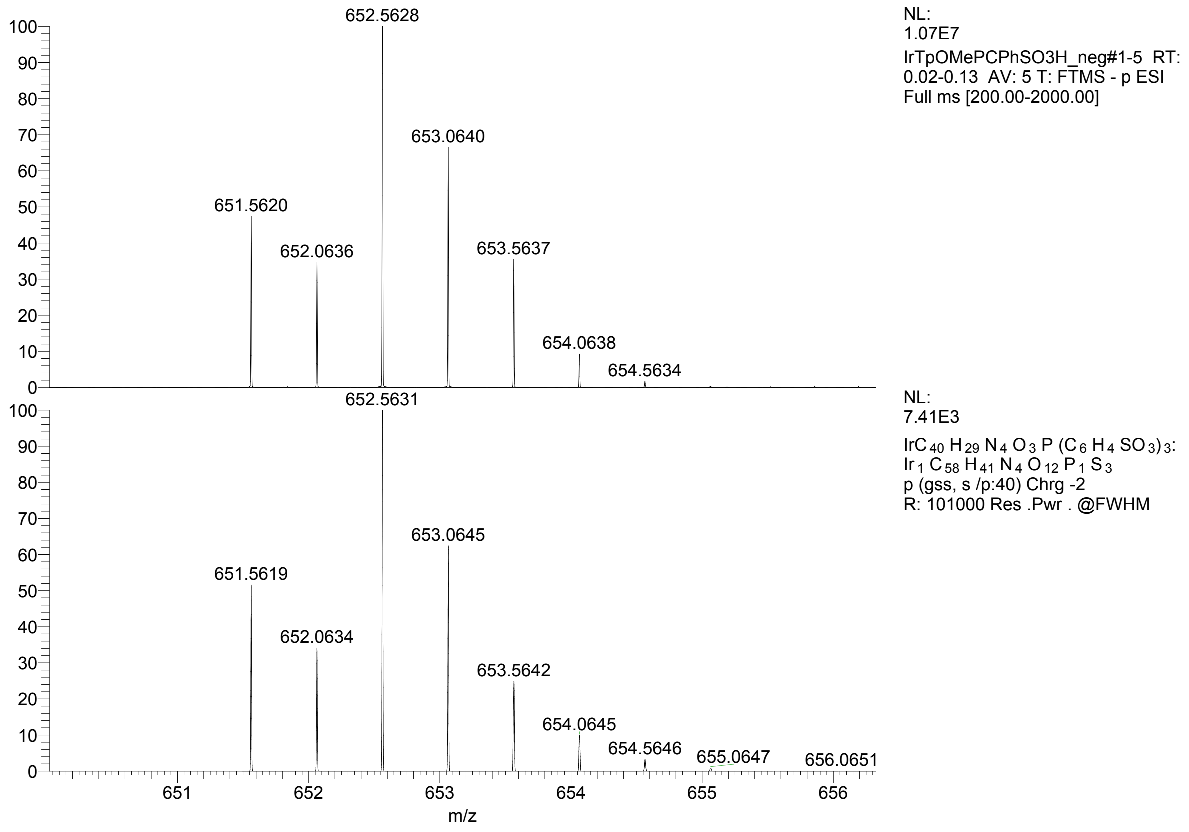

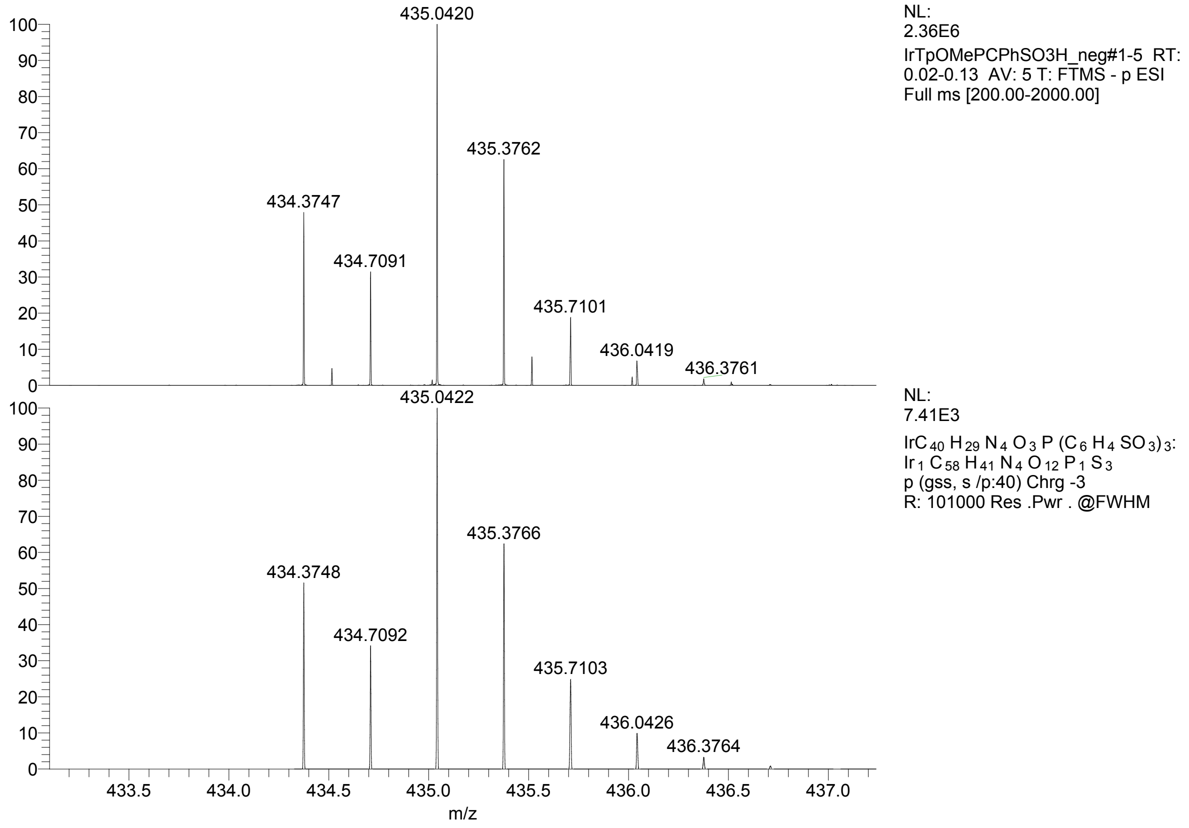

Figure S6 (contd.). Negative-mode ESI mass spectra of Ir[T*p*OMePC](tppts) (Z=2 and 3).

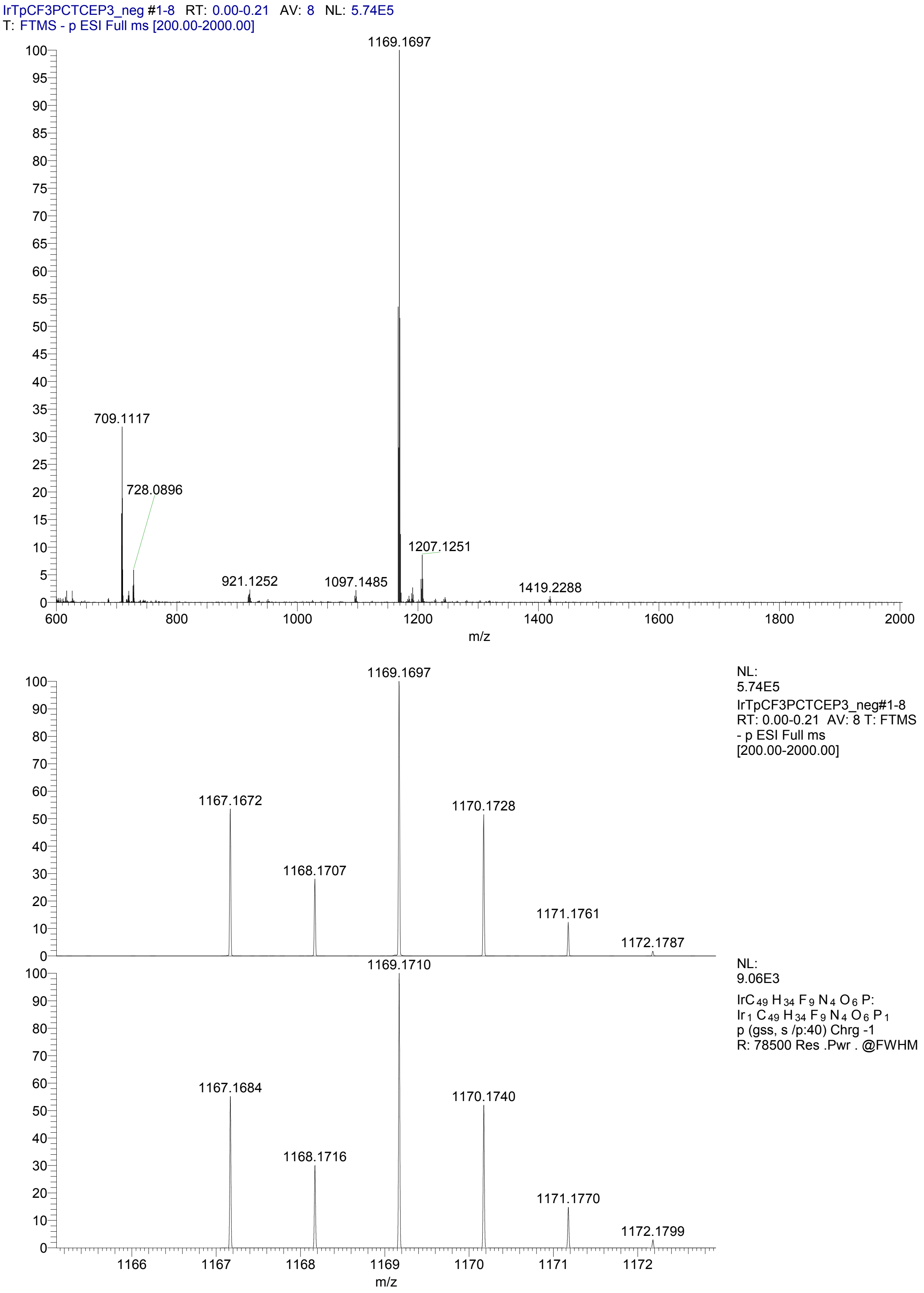

Figure S7. Negative-mode ESI mass spectra of Ir[(T*p*CF_3PC)](tcep).

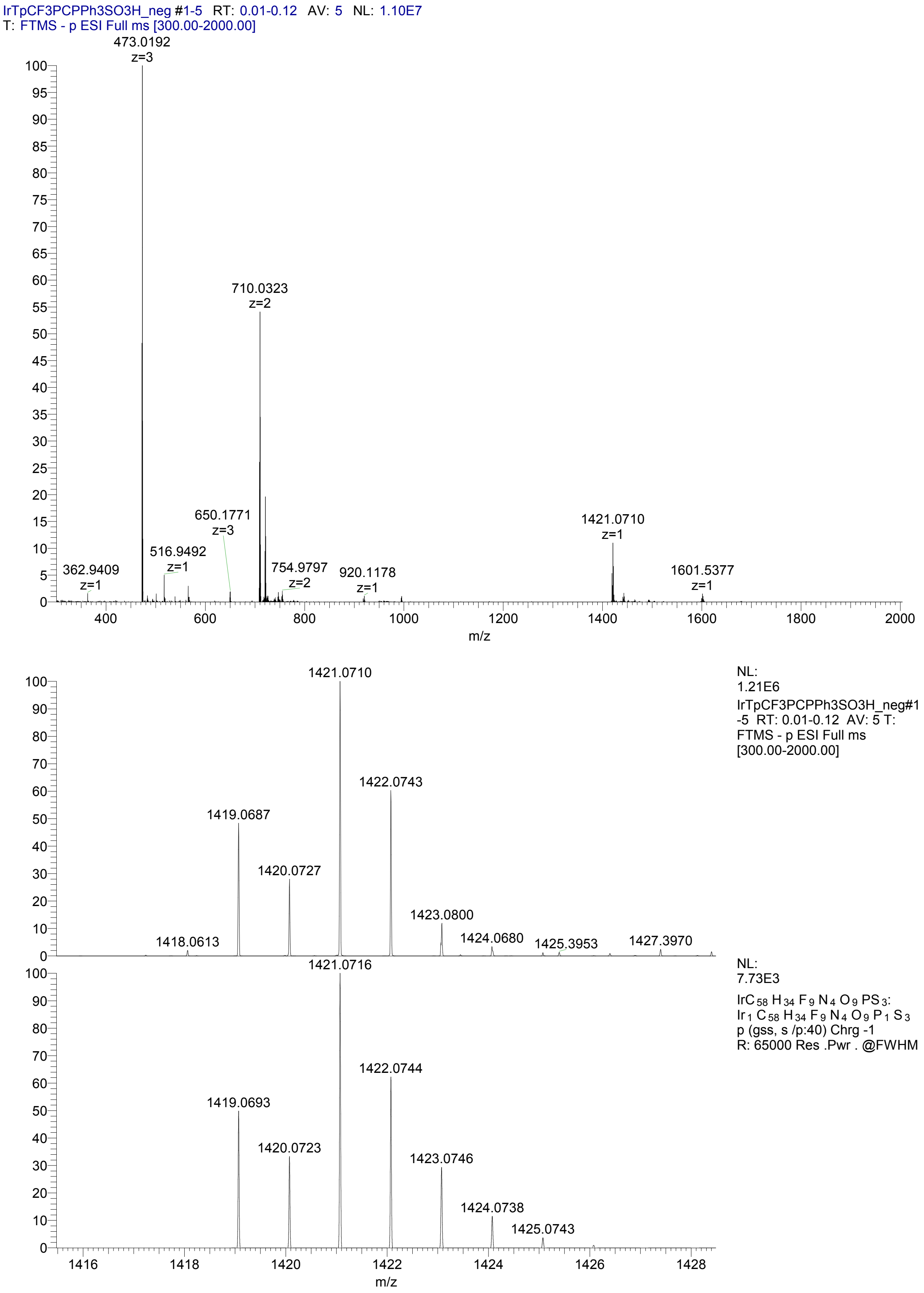

Figure S8. Negative-mode ESI mass spectra of Ir[T*p*CF$_3$PC](tppts) (Z=1). *Contd.*

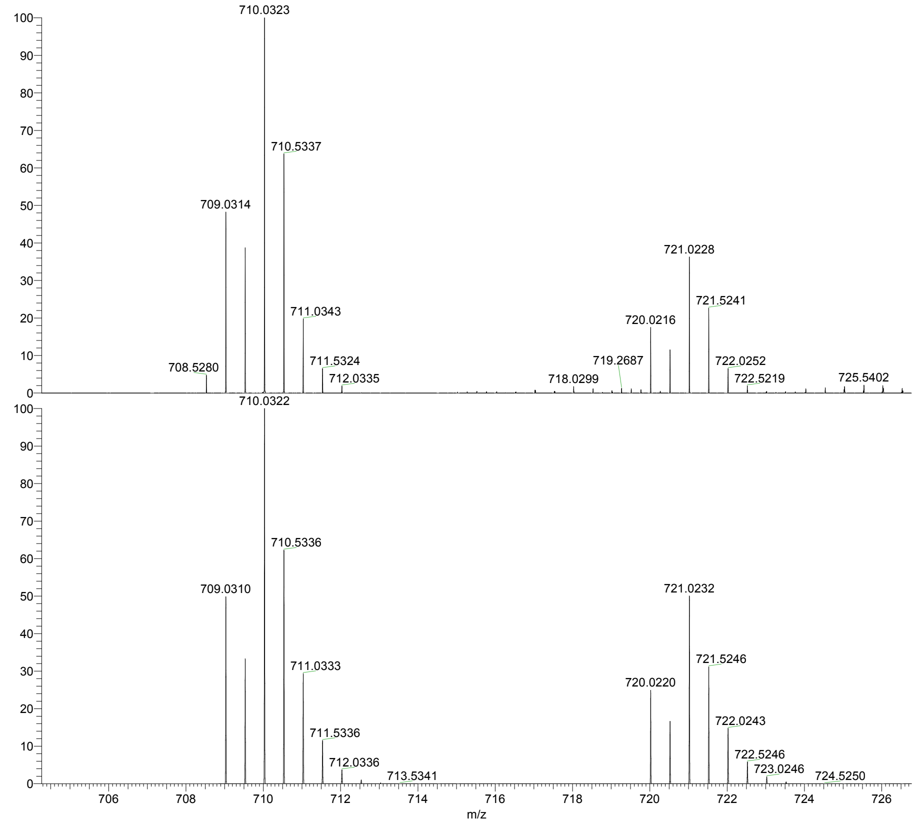

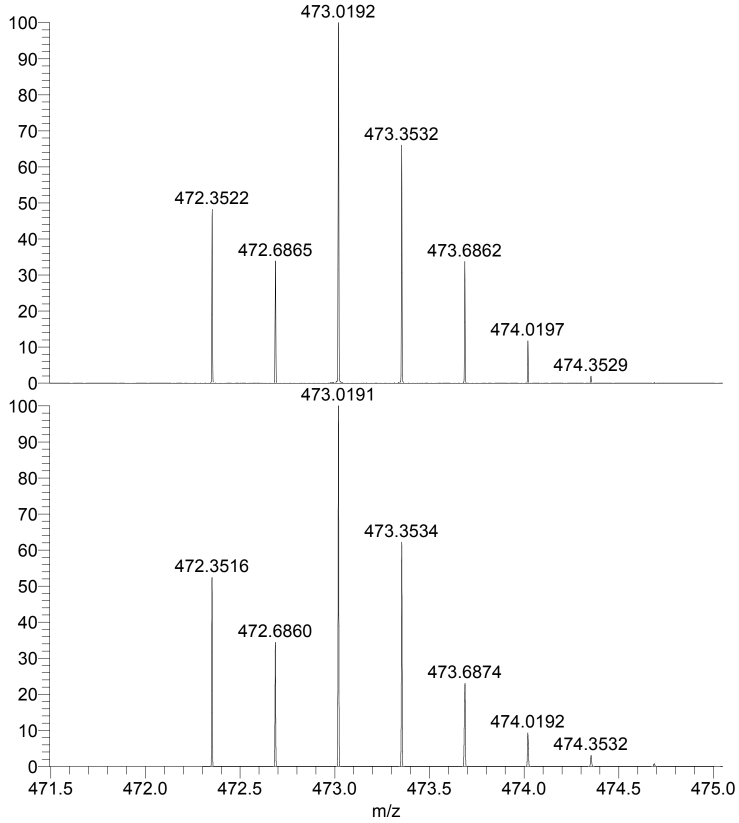

Figure S8 (contd.). Negative-mode ESI mass spectra of Ir[T*p*CF_3PC](tppts) (Z=2 and 3).

C. NMR spectra

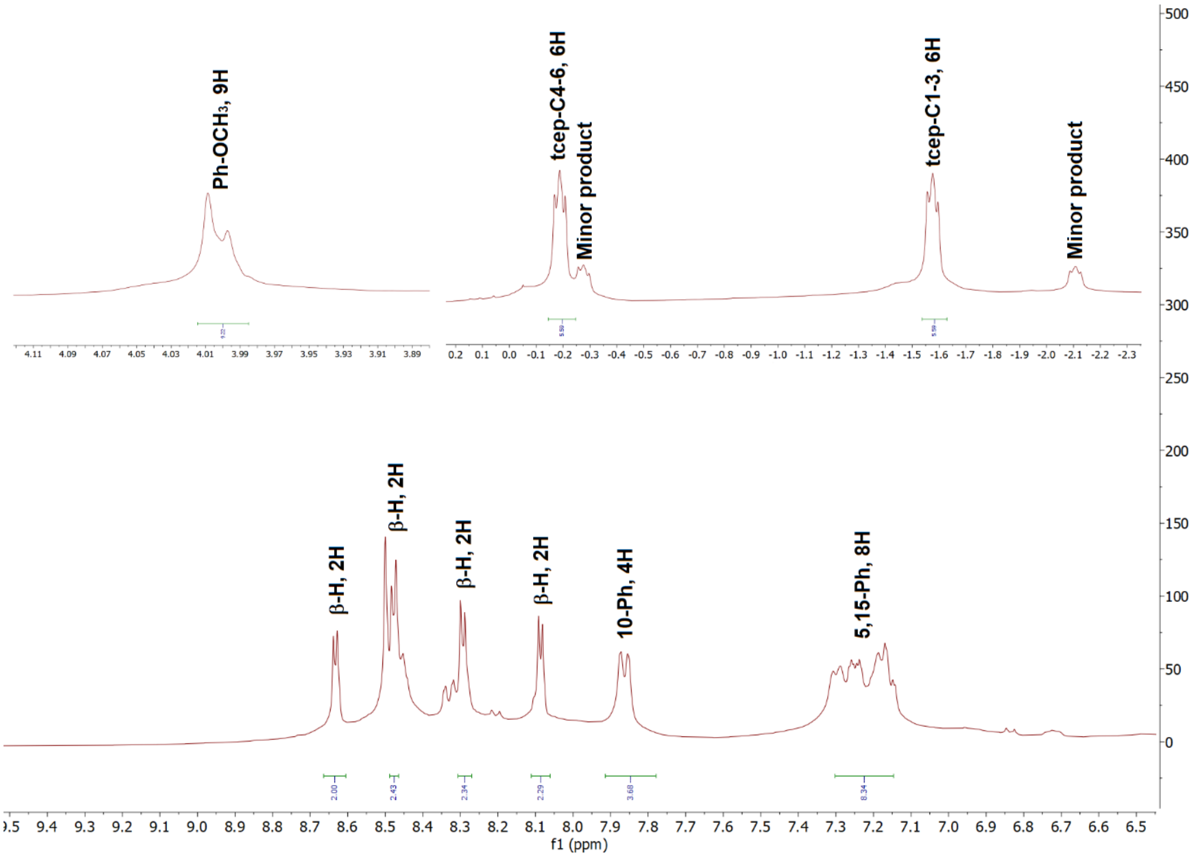

Figure S9. ^{1}H NMR spectrum of Ir[T*p*OMePC](tcep) in methanol-d_4.

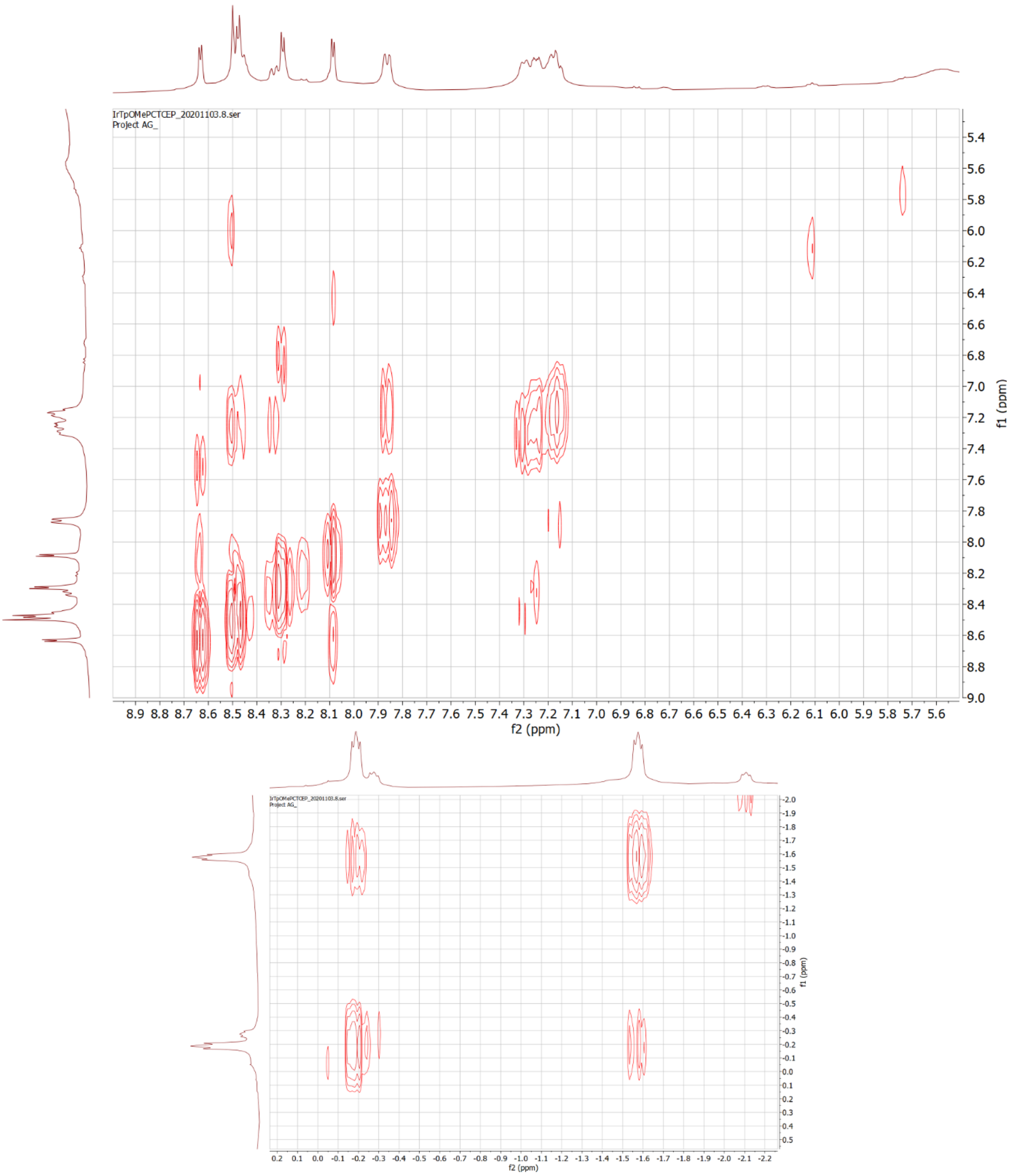

Figure S10. ^{1}H-^{1}H COSY of Ir[T*p*OMePC](tcep) in methanol-*d*$_4$. Top: close-up of aromatic area; bottom: close-up of ligand area.

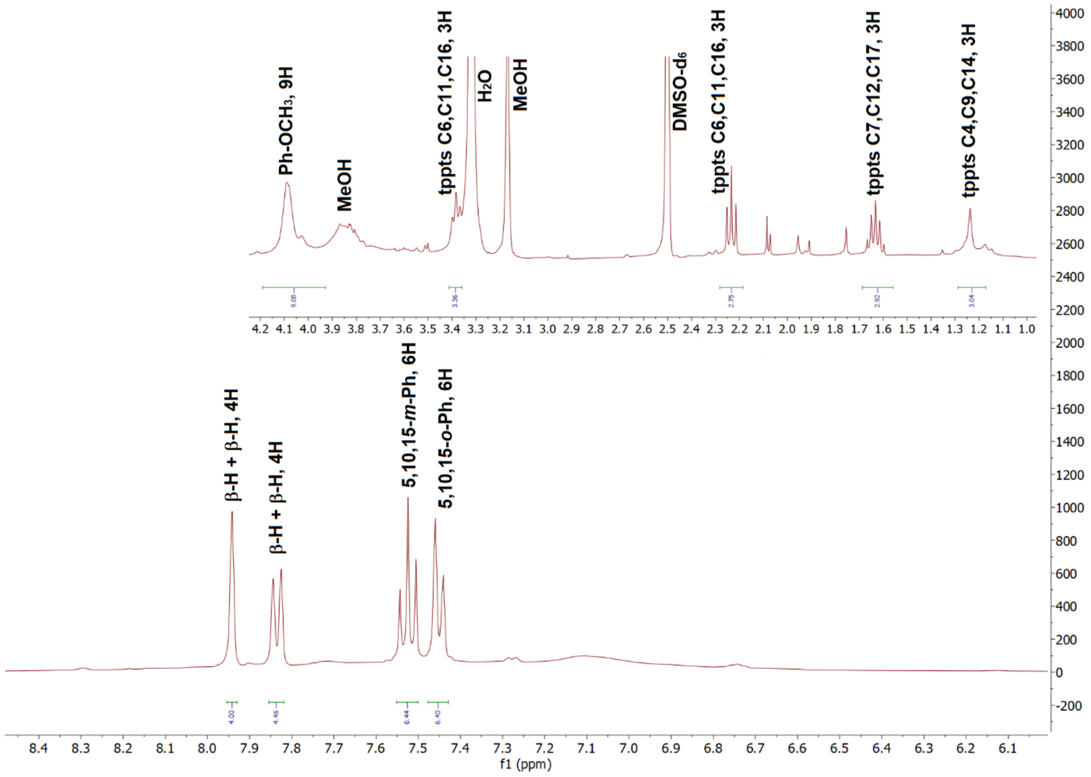

Figure S11. ^{1}H NMR spectrum of Ir[T*p*OMePC](tppts) in DMSO-*d*$_6$.

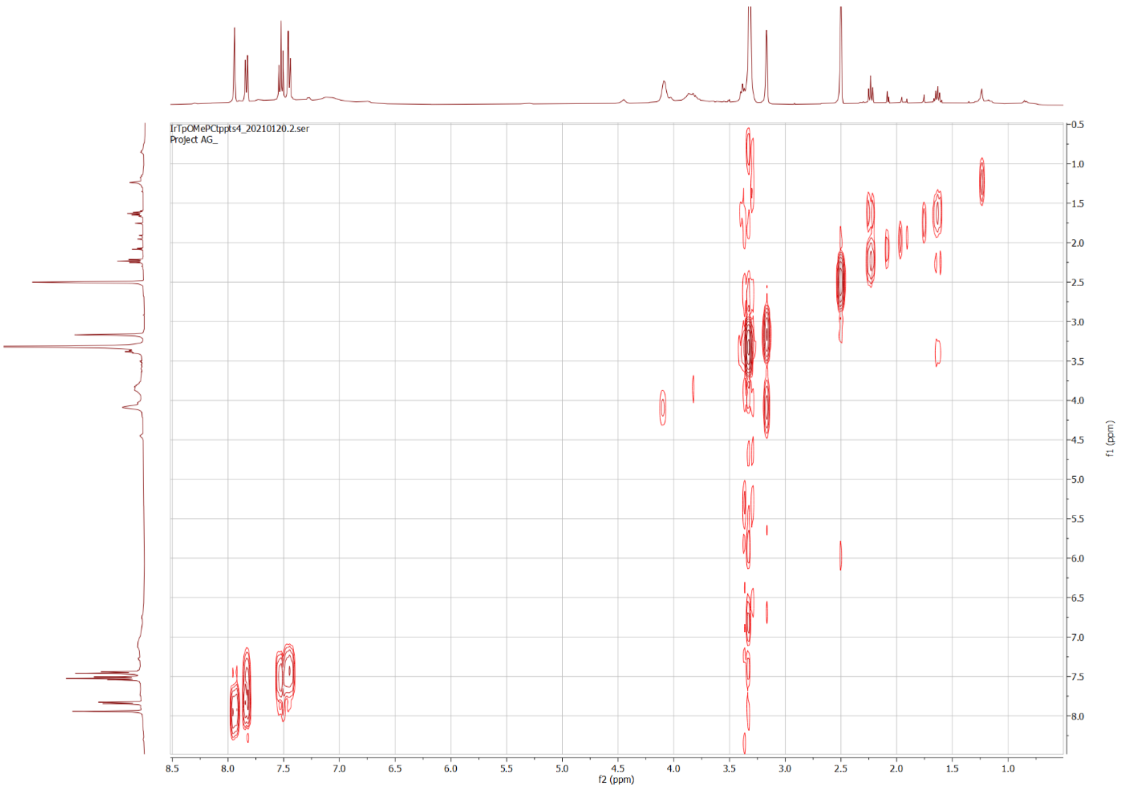

Figure S12. ^{1}H-^{1}H COSY of Ir[T*p*OMePC](tppts) in DMSO-*d*$_6$.

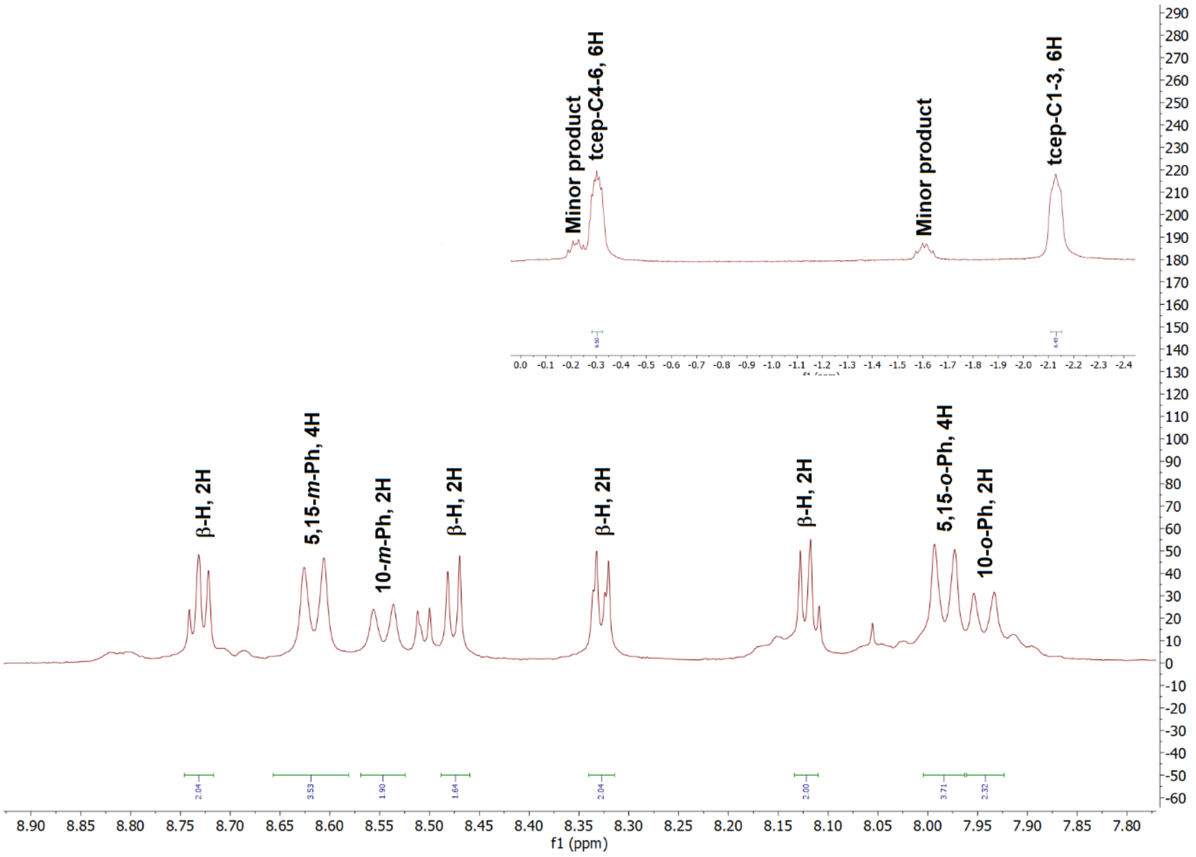

Figure S13. ^{1}H NMR spectrum of Ir[T*p*CF_3PC](tcep) in methanol-*d*$_4$.

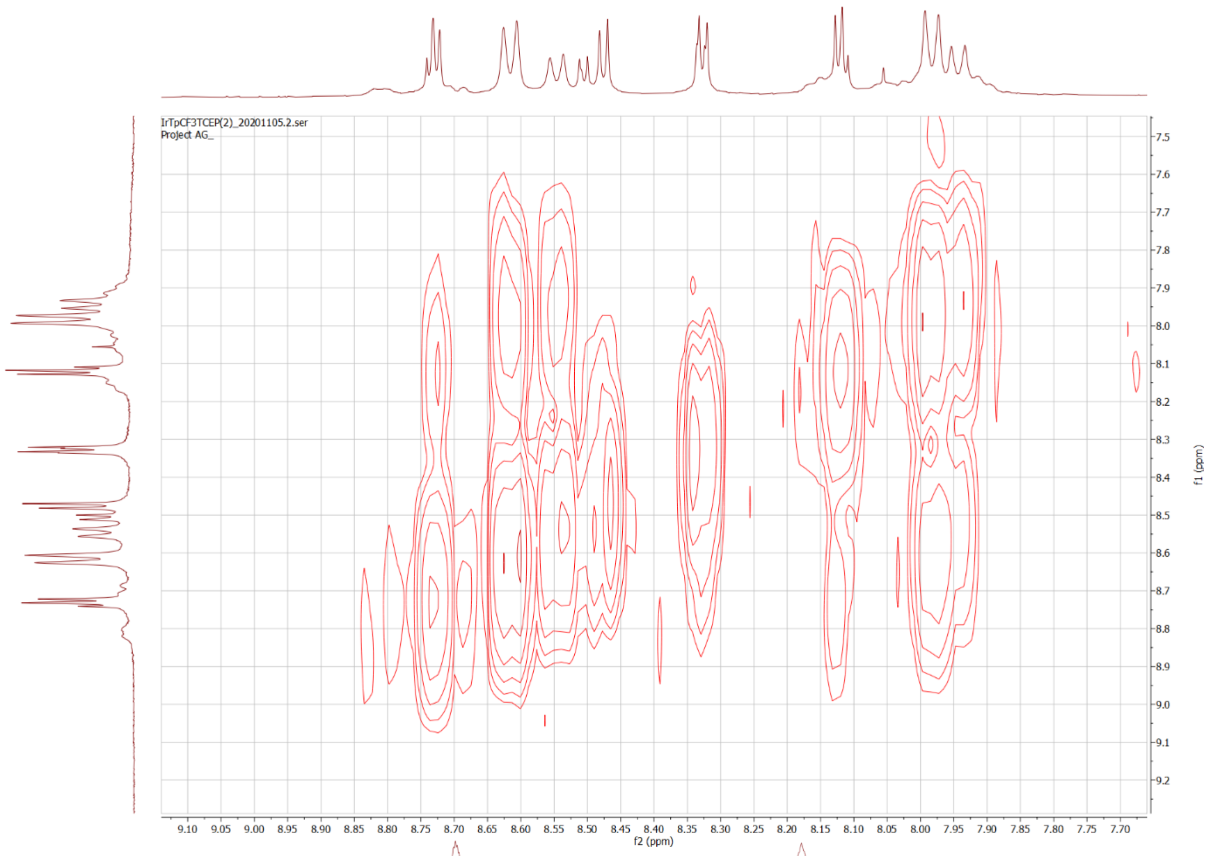

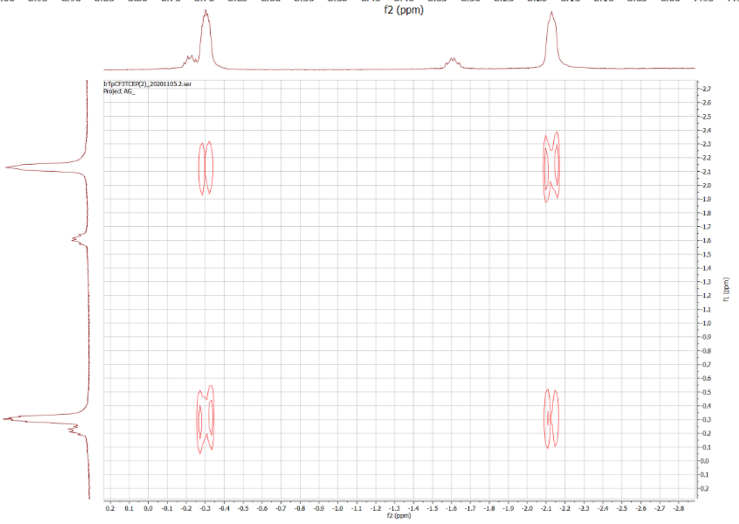

Figure S14. ^{1}H-^{1}H COSY of Ir[T*p*CF$_3$PC](tcep) in methanol-*d*$_4$. Top: close-up of aromatic area; bottom: close-up of ligand area.

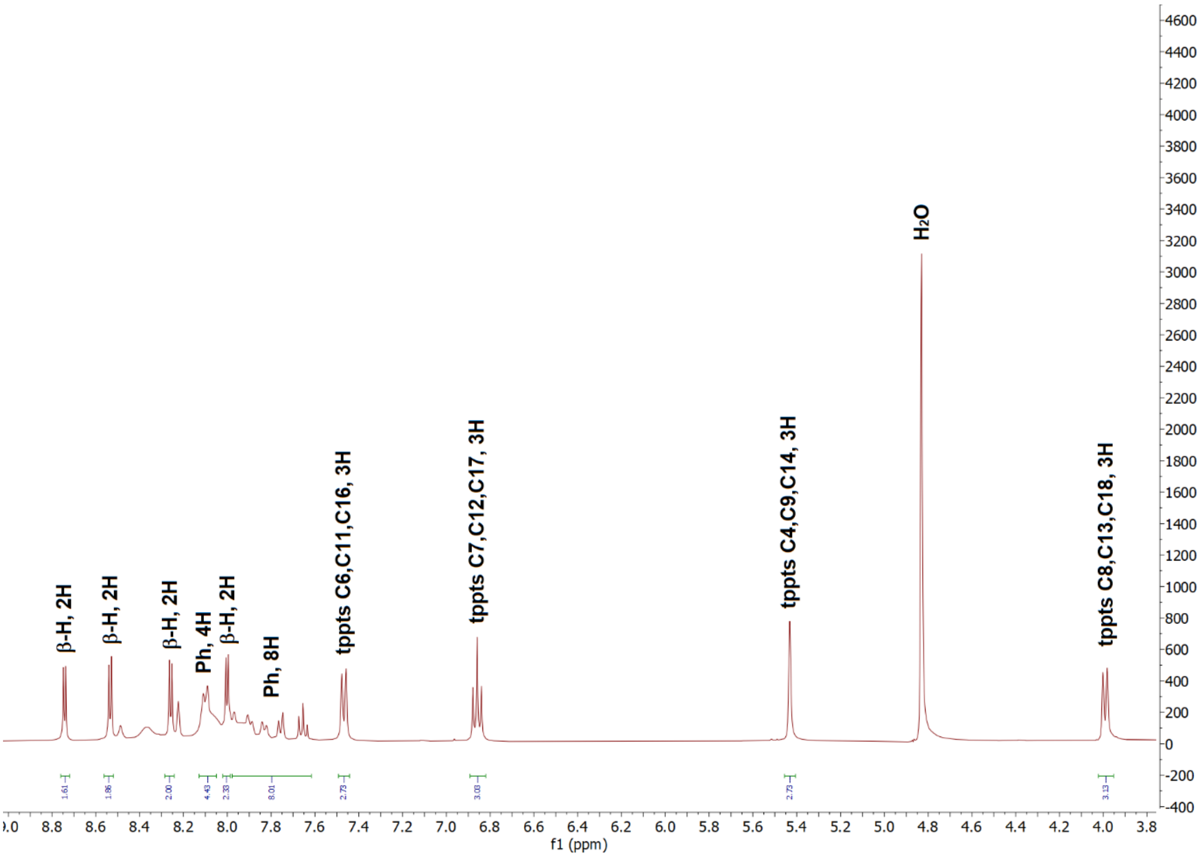

Figure S15. ^{1}H NMR spectrum of Ir[T*p*CF$_3$PC](tppts) in methanol-*d*$_4$.

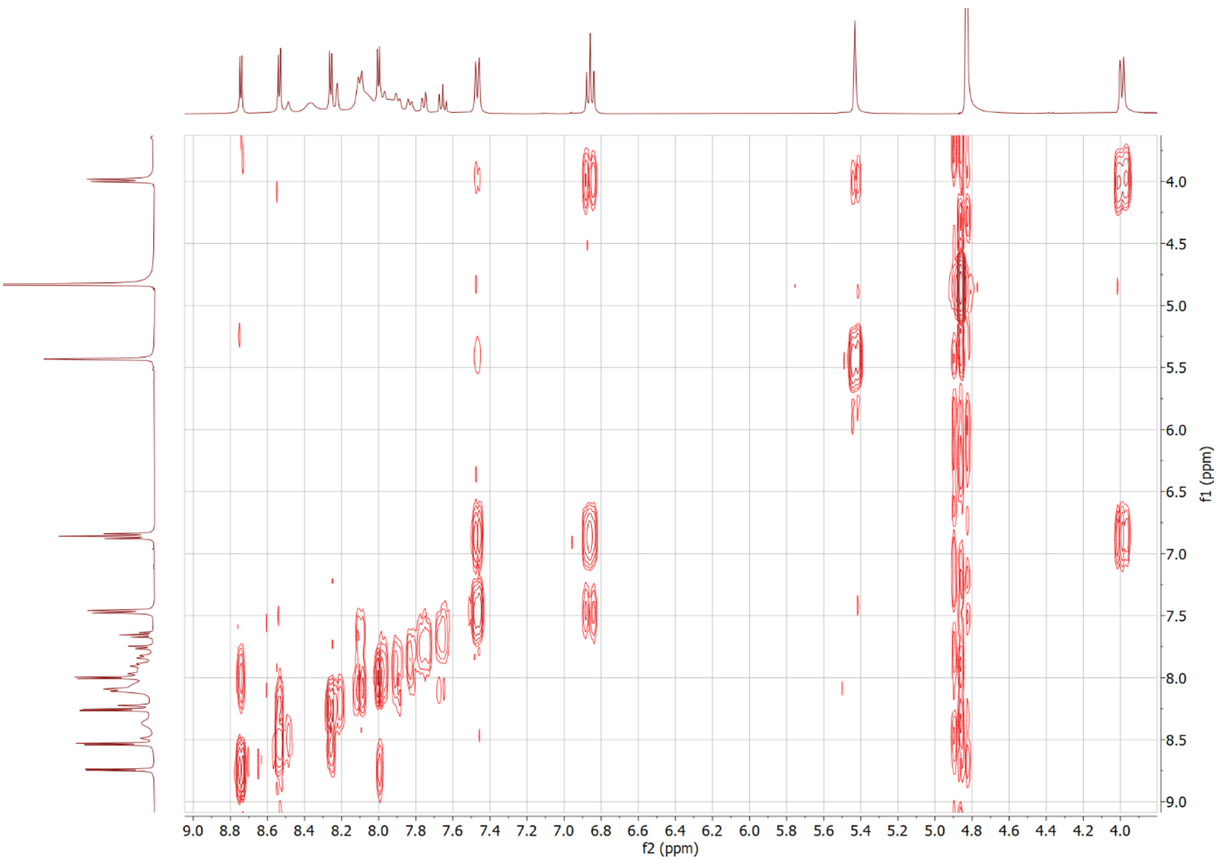

Figure S16. ^{1}H-^{1}H COSY of Ir[T*p*CF_3PC](tppts) in methanol-d_4.
